# Auricular Acupressure on Specific Points for Hemodialysis Patients with Insomnia: A Pilot Randomized Controlled Trial

**DOI:** 10.1371/journal.pone.0122724

**Published:** 2015-04-15

**Authors:** Chuan Zou, Lihong Yang, Yuchi Wu, Guobin Su, Shuhui Chen, Xinfeng Guo, Xiuqing Wu, Xusheng Liu, Qizhan Lin

**Affiliations:** 1 Nephrology Center, Guangdong Provincial Hospital of Chinese Medicine, Guangzhou, Guangdong province, P.R. China; 2 Evidence-based Medicine and Clinical Research Service Group, Guangdong Provincial Hospital of Chinese Medicine (The Second Affiliated Hospital/Clinical College, Guangzhou University of Chinese Medicine, Guangdong Provincial Academy of Chinese Medical Sciences), Guangzhou, Guangdong province, P.R. China; Medical University of Graz, AUSTRIA

## Abstract

**Objectives:**

To assess the feasibility and acceptability of a randomized controlled trial compared auricular acupressure (AA) on specific acupoints with AA on non-specific acupoints for treating maintenance hemodialysis (MHD) patients with insomnia.

**Methods:**

Sixty three (63) eligible subjects were randomly assigned into either AA group received AA on specific acupoints (n=32), or sham AA (SAA) group received AA on points irrelevant to insomnia treatment (n=31) for eight weeks. All participants were followed up for 12 weeks after treatments. The primary outcome was clinical response at eight weeks after randomization, defined as a reduction of Pittsburgh Sleep Quality Index (PSQI) global score by 3 points and more.

**Results:**

Fifty-eight (58) participants completed the trial and five dropped out. Twenty participants in AA group (62.5%) and ten in SAA group (32.3%) responded to the eight-week interventions (χ^2^ = 5.77, *P* = 0.02). PSQI global score declined 3.75 ± 4.36 (95%CI -5.32, -2.18) and 2.26 ± 3.89 (95%CI -3.68, -0.83) in AA group and SAA group respectively. Three participants died during the follow-up period. No evidence supported their deaths were related to the AA intervention. No other adverse event was observed.

**Conclusion:**

Feasibility and logistics of patient recruitment, randomization procedure, blinding approach, interventions application and outcome assessment had been tested in this pilot trial. The preliminary data appeared to show a favorable result on AA treatment. A full-scale trial is warranted.

**Trial Registration:**

Chinese Clinical Trial Registry ChiCTR-TRC-12002272.

## Introduction

Sleep problems are among the most frequent complaints in dialysis units. Approximately 50–80% of patients with end-stage renal disease (ESRD) complain of insomnia [1–4], which is nearly two-fold more than general populations do [5]. Previous researches have showed that poor sleep quality is linked to the duration of disability, high health-care utilization, poor quality of life and mortality in maintenance hemodialysis (MHD) patients [6,7]. Self-reported sleep quality in MHD patients is associated with hypertension and non-dipping blood pressure [8,9], indicating that poor sleep may be one of the important factors contributing to the high prevalence of cardiovascular events observed in MHD patients.

The etiology of insomnia in patients with chronic kidney disease (CKD) is multifactorial and complicated. Pathophysiologic abnormalities, psychological problems, lifestyle, and treatment-related factors lead to this sleep problem. Pathophysiologic conditions include anemia [2,10], hypoproteinemia [11], hyperphosphatemia [2,10], secondary hyperparathyroidism [2,12], micro-inflammatory state [10,11,13], and imbalance of autonomic nervous activity [14]. Psychological factors, like anxiety and depression [4] and uremic symptoms including limb pain [15] and pruritus [12] also play a role in some individuals. Prevalence of sleep problems decreases after renal transplantation [16,17], daily nocturnal dialysis [18] and parathyroidectomy [19].

Until now, only a limited number of pharmacologic studies and even less non-pharmacologic studies have focused on the treatment of insomnia in CKD patients. Consequently, treatment suggestions are mainly based on results obtained from non-CKD populations [20]. Benzodiazepine receptor agonists are the only agents currently approved by the US Food and Drug Administration (FDA) for treating insomnia. Although insomnia is often a chronic condition, the US FDA has only approved eszopiclone for clinical use without a specified time limit. Other sleep medications have been approved to use no longer than 35 days. The limited duration on prescription of these medications is due to the potential abuse, dependence and adverse effects, such as residual daytime sedation, cognitive impairment and poor motor co-ordination [21].

Owing to limitations of current treatment, many alternative therapies have been used to treat insomnia. In China, a country with a long history of applying Meridian Theory in health care, acupuncture, auricular acupuncture and auricular acupressure are common alternative therapies for insomnia. Auricular therapy, originally derived from auricular acupuncture, involves stimulation at specific acupoints on the outer ear with needles, pellets (*Semen vaccariae*), magnetic pearls and electricity [22]. Auricular acupressure (AA) is one of the typical types applied in diverse clinical conditions including sleep problems. Our preliminary work showed that AA adding to basic care (including sleep hygiene and sleep medications if necessary) achieved improvements on sleep quality, efficacy and duration in hemodialysis patients with severe insomnia [23]. We also found that patients consumed less sleep medications after receiving AA treatment. However, a recent meta-analysis of randomized control trials (RCT) and quasi-RCTs compared AA with sham control for insomniacs showed equivocal results [24]. Therefore, we planned to design a sham-control double-blind RCT to assess the efficacy and safety of AA for insomnia in the dialysis population.

Before conducting a full-scale RCT, some critical issues regarding feasibility assessment should be evaluated by performing a pilot study. Consequently, we conducted a pilot trial compared genuine AA with sham AA (SAA) presenting as AA on non-specific acupoints for MHD patients with insomnia, which aimed to assess the feasibility of recruitment, randomization, allocation concealment, blinding, intervention protocols, outcome assessment procedures and acceptability of AA treatment.

## Methods

The protocol for this trial and supporting CONSORT and STRICTA checklists are available as supporting information; see S1 CONSORT Checklist, S2 STRICTA Checklist and S1 Protocol.

### Study design

This pilot study was designed to be a two-arm (allocation ratio 1:1), double-blind, sham-controlled, randomized trial, and registered on Chinese Clinical Trial Registry (ChiCTR-TRC-12002272).

### Ethics approval

This trial was carried out according to the principles of the Declaration of Helsinki (Version Edinburgh 2000). The study protocol and written informed consent were approved by Institutional Ethics Review Boards of Guangdong Provincial Hospital of Chinese Medicine (B2011-28-01). All participants provided written informed consent.

### Participants

Participants were recruited through invitation letters, information pamphlets and posters in Hemodialysis Center of Guangdong Provincial Hospital of Chinese Medicine (Guangzhou, P.R. China). Patients who met all of the following inclusion criteria were invited to participate in the trial.

#### Inclusion criteria

- Under regular maintain hemodialysis treatment (weekly treatment hour ≥10) for 12 to 120 months;
- Aged 18~75 years;
- Chronic primary insomnia diagnosed according to Diagnostic and Statistical Manual of Mental Disorders fourth edition- Text Revision (DSM-IV-TR) [25];
- Global score of Pittsburgh sleep quality index (PSQI) > 7;
- Free of hypnotics use or using a minimum maintaining dose of estazolam (≤ 1mg/d) during the past three months;
- Informed consent provided.

#### Exclusion criteria

Those with any of the following conditions were excluded.

- Presence of co-morbidities including cancer, congestive heart failure, connective tissue disease and hematologic diseases;
- Inadequately dialyzed, indicating by urea clearance index (Kt/V) < 1.20;
- Presence of severe physical symptoms such as bone pain, itchy skin, sleep apnea and restless legs which are obviously causative for insomnia, and fatigue caused by severe anemia (hemoglobin < 60g/L) or malnutrition (serum albumin < 30g/L).

### Interventions

**Fig 1:**
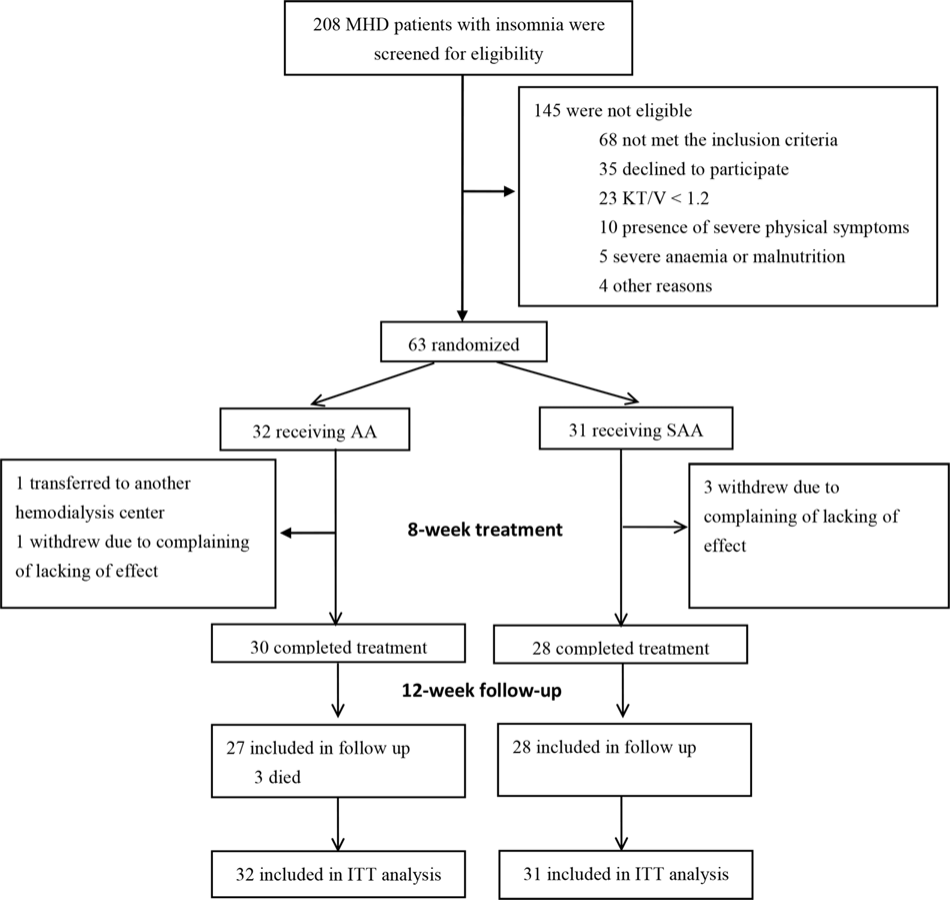
Flow chart of participants enrollment, assignment and follow-up.

This trial included an eight-week treatment period and a 12-week post-treatment follow-up period. Eligible participants were randomly assigned to receive AA either on specific acupoints or on sham acupoints (Fig 1).

#### Acupoints used in AA group

**Fig 2:**
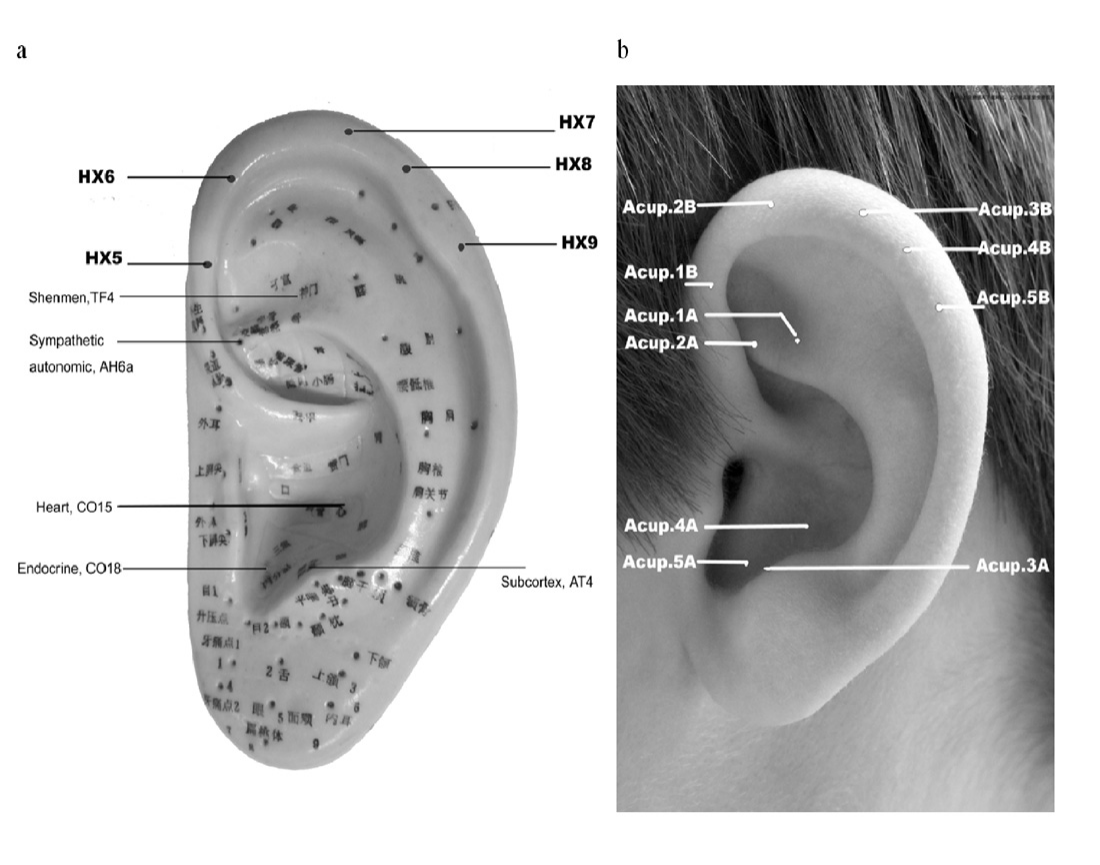
Auricular acupoints. a: Auricular acupoints used in treatment and control group. b: Codes for the acupoints used.

Participants in AA group received AA on five active acupoints including *Shen men* (TF4), *Sympathetic autonomic* (AH6a), *Subcortex* (AT4), *Heart* (CO15), and *Endocrine* (CO18), as illustrated in Fig 2.

#### Acupoints used in SAA group

Participants in SAA group received AA on five Helix points (HX 5–9), which were clearly remote from the inner ear area. (Fig 2)

#### Standard procedure for AA manipulation

AA was provided when participants were on hemodialysis in a state of relaxation. Both interventions were delivered by a trained nurse practitioner (Z.C. Zhong), who had no prior experience on AA before and was unaware of the group allocation. Acupoint areas were disinfected with 75% isopropyl alcohol before attaching a 1.0cm × 1.0cm adhesive plaster with one bead (*Semen Vaccariae*, globes of around 2.0mm in diameter; surface: smooth; color: black; Taicheng Technology & Development co., LTD, Shanghai, China) imbedded. Participants were instructed by the trained nurse to press the beads with continuous and appropriate finger strength in three to four kg and with the rate of one to two per second until a hot sensation and slight soreness on the points were felt [26]. Each acupoint should be manipulated three to five times in the daytime and evening. We did not recommend pressing at late night, especially one hour before going to sleep. The plasters were superseded every two to three days (usually on their dialysis day) and acupoints on two ears were used alternately. If the plasters or beads detached, the patients were asked to come to hospital to receive fresh plasters.

### Rationale for acupoint selection

Auricular acupoints were selected because of the following reasons: (1) According to traditional Chinese medicine (TCM) pathology of insomnia [27], treatments should focus on regulating the imbalanced *yin* and *yang* of the Heart. *Shen men* (TF4) and *Heart* (CO15) are related to the Heart and able to calm the spirit. The other acupoints help self-modulating the functions of internal organs. (2) Hyperarousal is deemed to be a pathophysiological factor in insomnia [28,29]. Acupoints locating at the triangular fossa and cavum conchae are likely to stimulate the vagus nerve, thus modulating autonomic nervous system (ANS) [30,31]. (3) A prescription of AA contained acupiont TF4, AT4, AH6a, CO15 and CO18 had been applied to insomnic MHD patients. Encouraging results was obtained in our preliminary study [23].

### Outcome measurements

#### Feasibility

Feasibility of this trial was assessed by the percentage of recruitment, retention, attendance and adherence.

#### Effectiveness

Sleep quality was characterized with the Chinese version of PSQI [32]. PSQI contains seven domains scoring from 0 to 3, with a total score ranging from 0 to 21. The higher score indicates the worse sleep quality. The permission of using PSQI was obtained from Prof. Buysse via email. The reliability and validity of its Chinese version had been proved [33,34]. PSQI was assessed at baseline, 4, 8 weeks after randomization and 4, 8, 12 weeks post-treatment.

(1) Primary outcome

The primary outcome was the clinical response at eight weeks after randomization. Response was defined as a reduction of PSQI global score by 3 points and more according to literature review [35].

(2) Secondary outcomes

Secondary outcomes were PSQI global score, and scores of each domain including sleep duration, sleep disturbance, sleep latency, day dysfunction, sleep efficiency, overall sleep quality and sleep medication. All participants underwent blood biochemical tests before randomization and at the end of follow-up. Parameters including serum creatinine (SCr), blood urea nitrogen (BUN), total carbon dioxide (TCO_2_), potassium (K^+^), calcium (Ca^2+^), phosphate (PO_4_^3-^), parathyroid hormone (PTH), albumin (Alb), hemoglobin (Hb) and Kt/V were all essential for monitoring the complications for MHD patients and adequacy of dialysis. If participants required hypnotic agents during the study because of unbearable sleep disorders, they were allowed to take hypnotics initiating from the minimum dose and encouraged to complete the trial. The weekly dose of hypnotic agents was recorded. Adverse events throughout the treatment and follow-up periods were documented and dealt with by appropriate measures.

### Randomization and allocation concealment

A random sequence generated by Microsoft Excel software 2003, was produced by an investigator not involved in running the trial. The random sequence was kept by him and the assignment was unaware to other research staffs.

### Blinding

Both the participants and the research nurse were blinded to the treatment protocol. Because acupoint names in Chinese often reflect the effects of the point (e.g. *Shen men* refers to tranquilization), all points used in the study were given a code to avoid breaking the blinding (e.g. 1A, 1B, see Fig 2). The nurse selected for this study did not have previous AA training or experience. He was made aware that the study was going to compare two different AA protocols, and was asked not to read the AA chart during the study. He was trained to apply both treatment protocols, and instructed not to discuss the difference of treatments with the participants. To avoid the Hawthorne and Rosenthal effects, he was required to limit the interaction with the participants. He would not ask participants whether they felt sore when placing the seeds on the acupoints (a common practice for confirming the accuracy of point location). The participants would not ask him any question about the manipulation and report any results to him. Any question regarding manipulation was directed to another investigator (X.Q. Wu) whom was responsible for teaching the participants how to apply the squeezing method. All results were reported to the investigator (Y.C. Wu) whom was responsible for recording results in a clinical interview. Another investigator, who conducted the statistical analysis, did not know the group assignment until the analyses completed.

### Sample size

As this pilot study was an exploratory study and undertook for the feasibility, sample size calculation was not performed.

### Statistical analysis

A descriptive analysis was performed. Mean and standard deviations (SD) were calculated for the quantitative variables and frequency distributions for the qualitative ones. Outcome of clinical response was analyzed by *Chi*-squared test or Fisher’s exact. Repeated-measures analyses of variance were conducted to compare changes on PSQI global score and each domain. Limited to the nature of pilot study, there was not adequate power to detect statistical difference, and therefore data were only analyzed to indicate the direction of effect of AA treatment. All analyses were performed on the intention to treat (ITT). We assumed that the unobserved data were missing completely at random based on little’s MCAR test (χ^2^ = 18.03, *P* = 0.978). Imputation of missing data was carried out with the last observation carried forward (LOCF). The outcome values at eight weeks after treatment were the baseline values of the participants whom withdrew or dropped out. In this case, the withdrawals or dropouts were considered to be the ineffective cases, and it provided a conservative estimation. Statistical analysis was processed using the Predictive Analytics Software package (PASW Statistics 18).

## Results

Two hundred and eight (208) patients were screened for eligibility from September to November 2012. Of these, 35 (16.8%) declined to participate in the trial, 68 (32.7%) did not fulfill the inclusion criteria, 42 (20.2%) met the exclusion criteria. Eventually, 63 (30.3%) patients were included, with 32 randomly assigned to receive AA on specific acupoints and 31 on sham acupoints (Fig 1). Five participants (7.9%) withdrew during the study. Three participants in SAA group and one in AA group withdrew at four weeks after treatments, due to complaining of lacking in effect. One participant in AA group discontinued due to transferring to another hemodialysis unit (Fig 1).

As we applied interventions on their dialysis day, all participants except the withdrawals completed all treatment sessions and reported that they self-administered according to our instruction.

No significant difference was found between two groups on baseline demographic and clinical characteristics, including age, gender, co-morbidities, sleep medication intake, and dialysis adequacy (Table 1).

**Table 1:** Baseline Characteristic of Participants

	AA group(n = 32) Mean(SD)	SAA group(n = 31) Mean(SD)
Age(y)	53.28(12.68)	58.55(10.00)
Male sex (n, %)	17 (53.1)	11 (35.5)
Comorbidities (n, %)		
Diabetes	3(9.4)	6(19.4)
Hypertension	21(65.6)	21(67.7)
No comorbidities	8(25.0)	4(12.9)
Taking hypnotic (n, %)	11(34.4)	14(45.2)
Dialysis time per week (hour)	11.0(1.02)	11.10(1.01)
Serum Creatinine (μmol/L)	1114.22(211.92)	1048.61(297.53)
Blood urine nitrogen (mmol/L)	24.29(5.99)	22.15(6.35)
TCO_2_ (mmol/L)	19.95(3.32)	19.90(3.48)
Serum potassium (mmol/L)	4.74(1.14)	5.06(0.67)
Serum calcium (mmol/L)	2.19(0.24)	2.29(0.17)
Serum phosphorus (mmol/L)	2.20(0.60)	2.43(1.16)
PTH (pg/ml)	749.98(607.93)	978.22(666.23)
Albumin (g/L)	40.37(3.20)	39.62(2.89)
Hemoglobin (g/L)	112.47(12.03)	114.42(17.14)
KT/V	1.47(0.27)	1.44(0.28)

Abbreviations: TCO2: Total carbon dioxide content, PTH: Parathyroid hormone, KT/V: urea clearance index

**Fig 3:**
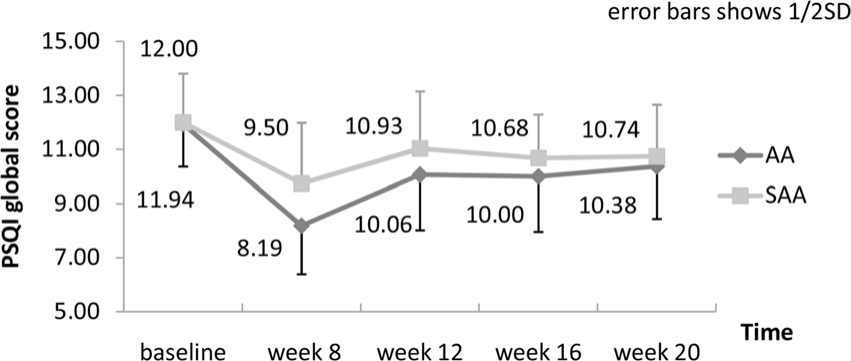
Change of PSQI global scores in two groups.

**Fig 4:**
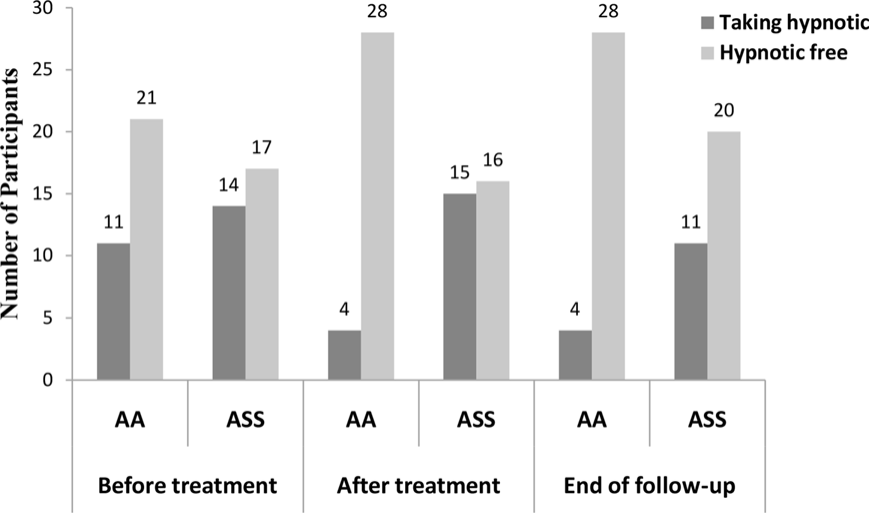
Numbers of participants taking hypnotic or hypnotic free. Before treatment between two groups (χ2 = 0.77, *P* = 0.38). After treatment between two groups (χ2 = 9.63, *P* < 0.01). End of follow-up between two groups (χ2 = 4.59, *P* = 0.03).

Missing data of five withdrawals were imputed with LOCF. Therefore, they were considered as non-response. Twenty (20) participants in AA group (62.5%) and ten (10) in SAA group (32.3%) responded to the eight-week interventions (χ^2^ = 5.77, *P* = 0.02). Changes of PSQI global score and of each domain within and between groups are presented in S1 Table. PSQI global score had decreased by 3.75 ± 4.36 (95%CI -5.32, -2.18) and 2.26 ± 3.89 (95%CI -3.68, -0.83) after treatment, and by 1.56 ± 3.66 (95%CI -1.15, -0.24) and 1.26 ± 2.97 (95%CI -2.35, -0.17) at the end of follow up, in AA group and SAA group respectively (see Fig 3). Result of repeated-measures analyses of variance for PSQI global score in Table 2 shows that PSQI global score declined over time both in AA group (F = 8.47, *P* < 0.01) and SAA group (F = 4.09, *P* < 0.01). Decrease also found in all seven domains except sleep disturbance after treatment in AA group. Decrease of day functional and use of sleep medications were maintained to the end of follow up in AA group (S1 Table). Number of participants taking sleep medications in AA group reduced from 11 (34.4%) to 4 (12.5%) after eight weeks of treatment which maintained to the end of follow-up, while the number in SAA group increased from 11 (35.5%) to 15 (48.4%) after treatment (χ^2^ = 9.63, *P* <0.01, Fig 4).

**Table 2:** Repeated-measures analyses of variance for PSQI global scores and its components over time in each group.

Outcomes	AA group (n = 32)		SAA group (n = 31)	
	F	*P*	F	*P*
PSQI score	8.47	0.00	4.09	0.00
Duration of sleep	4.39	0.00	2.37	0.06
Sleep disturbance	1.57	0.21	0.74	0.47
Sleep latency	4.37	0.00	1.75	0.14
Day dysfunctional due to sleepless	3.60	0.00	3.57	0.00
Sleep efficiency	2.11	0.08	2.30	0.06
Overall sleep quality	4.14	0.00	4.75	0.00
Use of sleep medications	5.25	0.00	1.20	0.31

Three participants died in AA group during the follow-up period, one because of myocardial infarction and two because of cardiac arrest. No evidence supported their deaths were related to AA intervention. No other adverse event was observed in both groups.

## Discussion

This pilot trial aimed to investigate the feasibility of a RCT compared AA on specific points with sham control for insomniacs in hemodialysis center. The trial was implemented smoothly in accordance with the study protocol.

Randomization procedures and allocation concealment were successful. All investigators except the one who generated and kept the random sequence were not aware of the group assignment. The approach of blinding of personnel and participants was feasible and effective in AA treatment trial. The research nurse would not be allowed to read the study protocol until the full-scale trial complete. Using non-specific auricular points as sham control was accepted. Helix (HX) points are assumed appropriately as non-specific points for insomnia, as they are clearly remote from the inner ear area and of little relevance with neither TCM theory on sleep treatment nor vagus nerve distribution [36].

In China, MHD patients are registered and regularly dialyze in outpatient hemodialysis center. Thus, we had the exact number of registered patients to screen for potential participants. Although the recruitment rate of this pilot study was just around 30%, recruitment was completed in a short period. Since all participants routinely dialyzed in our outpatient hemodialysis center and interventions were applied during their dialysis, all participants except the withdrawals completed the full treatment sessions.

Given the nature of a pilot study, we did not calculate the sample size, and therefore we had no adequate power to detect the statistical difference between two interventions. Nevertheless, encouraging indications were found from the results of the effectiveness outcomes. Our data showed, eight weeks after intervention, the response rate of AA group was almost two times higher than that of SAA group (62.5% vs. 32.3%). Besides, fewer participants in AA group than in SAA group were taking hypnotics (12.5% vs. 48.4%) after treatment, as well as at the end of follow-up (12.5% vs. 35.5%). Carryover effect of AA treatment for insomnia was observed, in terms of PSQI global score and the number of participant stopped taking hypnotics. This finding coincided with an observational study on the elderly with insomnia [37]. The results encouraged us to conduct a large full-scale RCT to evaluate the actual effect of AA in treating MHD patients with insomnia.

Based on the information gathered from this pilot trial, several essentials for conducting a further RCT properly were identified.

Withdrawal of hypnotics might be difficult. In view of the ethical issue and low compliance with the interventions, we recruited patients whom took a minimum dose of hypnotics (no more than one tablet of estazolam per night). About one-third participants took hypnotics before interventions in each group. Hypnotic agents somewhat contributed to the beneficial activity. In order to avoid this confounding, a three-arm trial that co-interventions were not allowed to use could be considered in further study design. A trial would be capable to compare AA with sham control, as well as an active control (hypnotics). Considering patients’ compliance, an inequality proportion of sample sizes also could be considered in sample size calculation, e.g. a proportion of 3:3:2 in AA group, SAA group and hypnotics group.

PSQI was the sole outcome measurement applied in the pilot trial. Combination with more specific insomnia questionnaire and measurements, such as insomnia Severity Index (ISI), sleeping diary could promote the reliability of the results.

As the full-scale study was planned to be a multi-center trial, a handbook of standard operating procedure would be necessary. Consistency in assessment is essential to reveal the actual effect. To pre-assess by outcome assessors from each center before the main trial, thereby delivering consistent outcomes.

Besides recruitment, patient retention and completed data are crucial to the success of a clinical trial and the validity of results. In the pilot trial, of five withdrawals, four complained of lacking in effect. Providing more educational material, thoroughly communication discussing the expectation prior to starting the trial, and reminding the value and importance of their contributions help to reduce the number of withdrawals.

## Conclusion

Feasibility and logistics of patient recruitment, randomization procedure, blinding approach, interventions application and outcome assessment had been tested in this pilot trial. The preliminary data appeared to show a favorable result on AA treatment. A modified full-scale trial is warranted.

## Supporting Information

S1 CONSORT Checklist (DOC)

S2 STRICTA Checklist (DOCX)

S1 Dataset Dataset of AA for MHD with insomnia trial. (XLS)

S1 Protocol Study protocol in English. (DOC)

S2 Protocol Study protocol in Chinese. (PDF)

S1 Table PSQI global score and its components of both groups. (DOC)
