# Supplementary material for: Auricular Acupressure on Specific Points for Hemodialysis Patients with Insomnia: A Pilot Randomized Controlled Trial
Source: PLoS One. 2015 Apr 15;10(4):e0122724. doi: 10.1371/journal.pone.0122724 (PMC4398355; doi:10.1371/journal.pone.0122724)
Supplement: S2 STRICTA Checklist — (DOCX) [file pone.0122724.s002.docx]

**Checklist for items in STRICTA 2010**

| **Item** | **Detail** | **Reported**  **on section** |
| --- | --- | --- |
| 1. Acupuncture rationale | 1a) Style of acupuncture (e.g. Traditional Chinese Medicine, Japanese, Korean, Western  medical, Five Element, ear acupuncture, etc) | Introduction  & interventions |
|  | 1b) Reasoning for treatment provided, based on historical context, literature sources, and/or  consensus methods, with references where  appropriate | Rationale for acupoint selection |
|  | 1c) Extent to which treatment was varied | - |
| 2. Details of needling | 2a) Number of needle insertions per subject per session (mean and range where relevant | Non-needle |
|  | 2b) Names (or location if no standard name) of  points used (uni/bilateral) | Interventions |
|  | 2c) Depth of insertion, based on a specified unit of measurement, or on a particular tissue level | Non-needle |
|  | 2d) Response sought (e.g. de qi or muscle twitch response) | Non-needle |
|  | 2e) Needle stimulation (e.g. manual, electrical) | Interventions |
|  | 2f) Needle retention time | Non-needle |
|  | 2g) Needle type (diameter, length, and manufacturer or material) | Non-needle |
| 3. Treatment regimen | 3a) Number of treatment sessions | Interventions |
|  | 3b) Frequency and duration of treatment  sessions | Interventions |
| 4. Other components of treatment | 4a) Details of other interventions administered to the acupuncture group (e.g. moxibustion, cupping, herbs, exercises, lifestyle advice) | - |
|  | 4b) Setting and context of treatment, including  instructions to practitioners, and information and explanations to patients | Interventions |
| 5. Practitioner background | 5) Description of participating acupuncturists  (qualification or professional affiliation, years in acupuncture practice, other relevant experience) | Interventions |
| 6. Control or comparator | 6a) Rationale for the control or comparator in the context of the research question, with sources | Rationale for acupoint selection |
|  | 6b) Precise description of the control or comparator. If sham acupuncture or any other type of acupuncture-like control is used, provide details as for Items 1 to 3 above. | Interventions & blinding |

Note: This checklist, which should be read in conjunction with the explanations of the STRICTA items, is designed to replace CONSORT 2010’s item 5 when reporting an acupuncture trial.
