# Supplementary material for: Auricular Acupressure on Specific Points for Hemodialysis Patients with Insomnia: A Pilot Randomized Controlled Trial
Source: PLoS One. 2015 Apr 15;10(4):e0122724. doi: 10.1371/journal.pone.0122724 (PMC4398355; doi:10.1371/journal.pone.0122724)
Supplement: S1 Protocol — (DOC) [file pone.0122724.s004.doc]

**Auricular Acupressure for Insomnia of Maintenance Hemodialysis Patients: Protocol of A Double-blinded, Randomized, Placebo controlled Pilot Study**

***1. Background and objectives***

Insomnia is one of the common complaints about sleep disorders of maintenance hemodialysis (MHD) patients. To get the desired sleep quality, hypnotic sedative agents are often prescribed at high doses and for long duration, raising risks of all kinds of adverse effects. As a non-drug therapy, auricular acupressure therapy (AA) is attractive to both patients and practitioners and is widely used to treat many conditions in China. However, concerning for its effectiveness in managing insomnia, its evidence base remains poor requiring rigorous high-quality trials.

In view of this, we designed this randomized controlled pilot study to test the hypothesis that auricular acupressure is a feasible and safe method treating insomnia for MHD patients, and to support carrying out rigorous large scale randomized controlled trials in the future.

***2. Flow chart of study procedures:***

Eligible MHD patients with insomnia

Week 1

AA treatment

SAA treatment

Week 8

Follow-up

Follow-up

Week 20

1. ***Study Design***

3.1 ***Study Design*** A double-blinded, randomized sham-controlled pilot study.

***3.2 Participants:*** MHD patients with insomnia.

***3.3 Inclusion criteria***

Subjects are selected MHD patients from local hemodialysis center (Hemodialysis center, Guangdong Provincial Hospital of Chinese Medicine) who are dialyzed twice or three times a week (total treatment time ≥ 10 hours per week). Those meeting all of the following criteria are included:

**·**Age 18~75 years

**·**Under regular maintain hemodialysis treatment for 12 and 120 months

**·**Chronic primary insomnia according to DSM-IV-TR，and PSQI global score > 7

**·**Independent of hypnotics, or using a minimal maintaining dose of hypnotics (e.g. estazolam≤1mg/d), during the last three months

**·**Informed consent.

***3.4 Exclusion criteria***

Patients with any of the following conditions are excluded:

**·**Presence of co-morbidities (e.g. cancer, congestive heart failure, connective tissue disease, hematologic diseases) and of psychiatric disorders;

**·**Inadequately dialyzed, indicating by urea clearance index (KT/V) lower than 1.20;

**·**Presence of severe physical symptoms such as bone pain and itchy skin, etc., which are obviously causative for insomnia;

**·**Fatigue caused by severe anemia (hemoglobin<60g/L) or malnutrition (serum albumin<30g/L).

***3.5 Randomization, allocation and Sample size***

A random sequence will be produced using computer software (Microsoft Excel 2003) and kept by an investigator who is not involved in running the trial. Eligible participants are randomly allocated into either AA group or Sham AA (SAA) group.

As a pilot study, sample size calculation was not performed. A sample size of 60 subjects is going to collect at a ratio of 1:1.

***6. Interventions***

***6.1 Co-intervention***

All patients maintain regular hemodialysis using dialysis machine from Fresenius Medical Care Co.Ltd. and B.Braun Co.Ltd. Reverse osmosis water is applied. For each treatment, we use disposable dialyzers with well biocompatibility and bicarbonate dialysate in a flow rate of 500ml/min. Blood flow rate is set at 180ml/min. Either unfractionated heparin (UFH) or low molecular weight heparin (LMWH) is used to prevent coagulation. Patients receive 2-3 hemodialysis sessions every week, each for 4 hours. Their Kt/V should be over 1.20, TACurea less than 17.9mmol/L，PCR more than 1.0 to1.2 and URR over 60％, indicating satisfactory dialysis sufficiency. Other agents such as anti-hypertensive drugs, erythropoietin, and iron supplements are administered according to related guidelines.

***6.2 Auricular acupressure intervention***

***Treatment group***

Participants in the treatment group will received AA on five active acupoints including

Acup.1A. *Shen Men* (*Spiritual Gate*, TF4),

Acup.2A. *Jiao Gan* (*Sympathetic autonomic*, AH6a),

Acup.3A. *Xin* (*Heart*, CO15),

Acup.4A. *Pi Zhi Xia* (*Subcortex,* AT4),

Acup.5A. *Nei Fen Mi* (*Endocrine*, CO18).

***Control group***

Participants in SAA group received auricular acupressure on Helix points (HX 5-9), which were clearly remote from the inner ear area.

Acup.1B. Gang Men (HX5)*,*

Acup.2B. Er Jian Qian (HX6),

Acup.3B. Er Jian Hou (HX7),

Acup.4B. Jie Jie (HX8),

Acup.5B. Lun yi (HX9).

***6.3 Standard procedure for AA manipulation***

AA is provided when participants are on hemodialysis in a relaxed status. Both interventions are delivered by one nurse who had no prior experience in AA. Each point is swabbed with 75% isopropyl alcohol before attaching a 1.0 cm × 1.0 cm adhesive plaster with one bead (*Semen Vaccariae*, globes of ~2.0 mm in diameter; surface: smooth; color: black; Taicheng Technology & Development co., LTD, Shanghai, China) imbedded. Subjects are instructed by the trained nurse to press the beads with continuous and appropriate finger strength for 1-2 minutes until a hot sensation and slight soreness on the points. Each acupoint should be manipulated three to five times in the daytime and evening. It is not recommended to press one hour before going to sleep. The plasters are replaced every 2-3 days (usually on their dialysis day) and acupoints on two ears are used alternately. If the plasters or beads detach, the patients should come to hospital to receive new plasters.

***7. Treatment duration and follow up duration***

Treatment period: 8 weeks

Follow up period: 12 weeks following end of treatment.

***8. Outcome measurements***

8.1 PSQI score: The sleep quality will be evaluated at baseline, end of treatment (week 8), 4 weeks (week 12), 8 weeks (week 16) and 12 weeks (week 20) after treatment.

8.2 Clinical and laboratory parameters [including serum creatinine (SCr), blood urea nitrogen (BUN), total carbon dioxide (TCO2), potassium (K+), calcium (Ca2+), phosphate (PO43-), parathyroid hormone (PTH), albumin (Alb), hemoglobin (Hb)]: at baseline, and at the end of follow-up (week 20).

8.3 The weekly dose of hypnotics

8.4 The clearance rate of urea nitrogen (Kt/V)

**9. *Patient safety***

Clinical and laboratory investigations and adverse events throughout the treatment and follow-up periods will be documented. Measures will be taken immediately to deal with potential adverse events such as fainting, severe pain and local infection, no matter whether they are related to AA. When necessary, the researcher will be informed to break the blindness.

**10. *Withdrawal and drop out***

All participants have a right to withdraw from the trial at any time. Any subjects who failed to complete the entire observation are drop-out cases, regardless of the time or reason.

Researchers will contact these patients and attempt to obtain additional information, including the reason for dropping out and the last time they receive treatment. If there were any correlation to adverse effect of drugs or treatments, it should be documented in case report form and reported to researchers. Withdrawal and drop out cases are also reported and analyzed.

***11. Statistical analysis***

Missing data will be imputed with the last observation. For dichotomous data, Chi-squared test or Fisher’s exact test will used. For continuous data, t test will be used. Repeated-measures analyses of variance will be conducted to compare changes on PSQI global score and each domain. P value less than 0.05 will be considered statistically significant. Statistical analysis will be processed using SPSS 18.0.

**12. *Blinding details***

All points used in the study are given a code in order to avoid revealing the differences between the two treatment protocols (1A-5A for AA group; 1B - 5B for SAA group). The nurse who had no prior experience on AA is trained to apply both treatment protocols. The researchers will tell him that the study uses two AA treatment protocols, but will not mention sham control. He will be required not to research the AA chart during the course of the study, and not to talk to participants about treatments.

Blinding will be kept for the investigator who is response for statistical analysis until all data analysis complete.

***13. Ethics consideration***

This trial will be performed according to the principles of the Declaration of Helsinki (Version Edinburgh 2000). Patients under observation in the trial will receive appropriate treatment and have no worry of delaying treatment or causing sufferings. The study protocol has been approved by regional ethics review boards of Guangdong Provincial Hospital of Chinese Medicine, with the permit number B2011-28-01. All participants enrolled should give their written informed consent.
