# Supplementary material for: Auricular Acupressure on Specific Points for Hemodialysis Patients with Insomnia: A Pilot Randomized Controlled Trial
Source: PLoS One. 2015 Apr 15;10(4):e0122724. doi: 10.1371/journal.pone.0122724 (PMC4398355; doi:10.1371/journal.pone.0122724)
Supplement: S2 Protocol — (PDF) [file pone.0122724.s005.pdf]

耳穴贴压法

对维持性血液透析患者睡眠障碍

的干预研究

研究者手册

项目承担单位: 广东省中医院透析科

研究计划开始日期：2012 年 4 月 01 日

研究计划结束日期：2012 年 12 月 30 日

编制时间：2011 年 03 月

第一版/2011.3

一、研究简介

随着透析技术的改进和患者存活时间的延长，提高血液透析患者的生活质量成为医护人员及患者的共同愿望。睡眠障碍在血液透析患者中发生率可高达60～80%，大部分患者因此需依赖精神类药物，长期服用可出现一定的副作用。中医理论对失眠有丰富的认识和独具特色的治疗，耳穴贴压就是在中医理论指导下在人体耳廓上某些特效穴位上施以按压刺激以调理治疗。我们拟进行一项初步的随机对照临床研究。

二、研究流程图

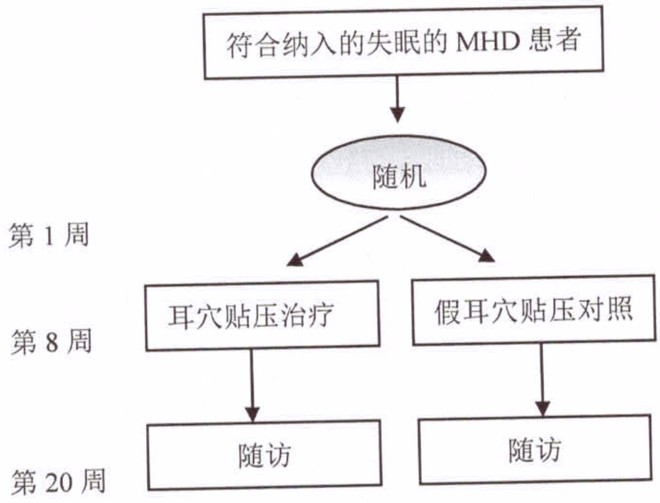

三、研究方案

1. 研究设计： 双盲随机对照临床试验。

2. 研究对象： 伴有睡眠的血液透析患者。

3. 纳入标准：

①年龄18～75岁；

②维持性血液透析12至120个月，规律透析每周2-3次（每周透析时间10h以上）；

③符合原发性失眠的诊断标准（DSM-IV-TR）且PSQI>7分；

④无使用催眠药物，或服用最小维持剂量（艾司唑仑≤1mg/d）3个月以上

⑤知情并同意者

4. 排除标准：

①合并严重的其它系统疾病（如肿瘤、充血性心衰、结缔组织病、血液病等）以及严重的精神疾病，如痴呆症等导致不能配合；

②透析不充分，KT/V 小于 1.20 者；

③明显是由于严重的躯体症状如骨痛、皮肤瘙痒等导致的失眠；

④严重的贫血（Hb＜60g/L）或营养不良（Alb＜30g/L）者。

5.分组与样本量

使用 Excel 2003 生成随机数字，结果专人管理，按入组顺序，将合格病例按 1:1 比例分为耳穴贴压治疗组和假耳穴贴压对照组。由于缺乏相关数据进行样本量估算，本预试验预计按 1:1 纳入总 60 例。

6.治疗措施

6.1 共同治疗措施

所有患者均采用费森尤斯或贝朗 Dialog 型透析机，透析用水为反渗水。透析时采用生物相容性好的一次性透析器，碳酸氢盐透析液，流速 500ml/min，血流量为 180ml/min，肝素或低分子肝素抗凝。每次透析 4h，每周透析 2-3 次，KT/V 大于 1.2，TACurea≤17.9mmol/L，PCR>1.0～1.2 及 URR>60%。治疗期间其他慢性肾衰药物如降压药，促红细胞生成素，铁剂等按原方案进行，纠正患者的营养不良、贫血及钙磷代谢紊乱等并发症。

6.2 耳穴取穴

耳穴贴压治疗组：神门、心、交感、皮质下、内分泌。

假耳穴贴压对照组：(耳轮的五个穴位)肛门，耳尖前，耳尖后，结节，轮 1

6.3 耳穴贴压操作

两组使用相同的操作方法：在充足的光线下，以左手固定耳廓，右手用探棒按压所取穴位，按压片刻，使用压痕作为压豆时的标记。将耳廓用 75%的酒精棉球进行消毒，待干(一则为了消毒，二则脱去耳廓上的皮脂，以利胶布贴紧，否则胶布易脱落)。以王不留行籽粘于 1.0 cm × 1.0 cm 的医用氧化锌胶布中点（上海泰成科技发展有限公司)，对准压痕贴敷好，并用适当的指力每穴按压约 1～2min，使之产生酸麻胀痛热感，耳廓表面红晕充血。嘱每日自行按压 3～5 次，刺激强度以患者感酸胀、麻木、灼热、能耐受为度，避免睡前一小时按压。每次选贴一侧耳穴，两耳交替贴压，隔 2-3 日换 1 次。如胶布脱落，嘱返院贴上新的。

注意事项：

① 防止胶布潮湿和污染，以免引起皮肤炎症。

②耳廓有皮肤损伤或冻疮处不宜贴压，以防感染。

③个别患者对胶布过敏，可改用防止过敏的纸胶布贴压。

7 治疗与随访时间

7.1 治疗时间：8 周

7.2 随访时间：治疗结束后随访 12 周

8.疗效评价

结局指标与相应的测量时间点：

- PSQI 匹兹堡睡眠质量评分：基线，治疗结束，随访 4 周、8 周、12 周
- 血液学检查（包括血肌酐，血尿素氮，总二氧化碳，血钾，血钙，血磷，甲状旁腺素，血清白蛋白，血红蛋白）：基线，随访结束后
- 每周催眠药物用药量，尿素氮清除率

9.安全性评估

实施治疗措施后，记录受试者主动提供或经非诱导性提问后得出的不良事件，以及临床和实验室检查的结果。并由医师对不良事件进行判断，对症处理，必要时终止试验，致电研究者进行破盲。并根据病情进行相关治疗，并记录。

10.退出、脱落的规定和处理：

所有签署了知情同意书并筛选合格进入试验的受试者，均有权随时退出临床试验，无论何时何因退出，只要没有完成临床试验全程观察，均为脱落病例。

退出、脱落病例的处理：当受试者脱落后，研究者必须在 CRF 中填写脱落原因，并尽可能与受试者联系，完成所能完成的评估项目。对因不良反应而脱落，经访视最后判断与试验药物有关者，必须记录在 CRF 中并上报课题组备案。退出、脱落病例应进行报告和分析。

11.统计分析：

所有缺失数据采用结转法（LOCF）进行填补。对基线数据，计量资料比较用 t 检验，计数资料采用卡方检验或精确检验。对 PSQI 使用重复测量数据的方差分析。$P<0.05$ 有统计学意义。采用 SPSS 18.0 统计软件包进行统计学分析。

12.盲法细节

所有选取的穴位均使用编码代号表示，试验过程中不提及穴位名称。耳穴贴压治疗组穴位编号为 1A-5A，假耳穴贴压对照组穴位编号为 1B-5B。

试验护士负责耳穴贴压的操作，人选必须没有针灸相关理论以及治疗操作经验。课题组仅告知该护士，本研究使用两种耳穴贴压方案治疗，不提及假耳穴的敏感字眼。要求该护士在研究完成全程观察之前不能翻阅研究方案，并且在操作过程中避免与患者就治疗方案进行交流。

在统计分析人员完成所有数据分析后对其揭盲。

13. 研究相关的伦理

本研究中进入观察的研究对象均给予相应治疗，不会延误病人的治疗，不会增加病人的身体痛苦。临床研究方案得到医院伦理委员会批准（文件号B2011-28-01）。按照医院相关的知情同意 SOP 进行知情同意过程的操作，所有研究对象在研究开始之前均由研究人员介绍本研究的目的和方法等情况，征得患者同意并获得签署的知情同意书。
