# Supplementary material for: Auricular Acupressure on Specific Points for Hemodialysis Patients with Insomnia: A Pilot Randomized Controlled Trial
Source: PLoS One. 2015 Apr 15;10(4):e0122724. doi: 10.1371/journal.pone.0122724 (PMC4398355; doi:10.1371/journal.pone.0122724)
Supplement: S1 Table — (DOC) [file pone.0122724.s006.doc]

**S1 Table**. PSQI global score and its components of both groups.

|  | **AA group (n=32), Mean(SD)** | Compared with baseline(*P* value) | **SAA group (n=31), Mean(SD)** | Compared with baseline(*P* value) | Factor* groups | |
| --- | --- | --- | --- | --- | --- | --- |
|  |  |  |  |  | F | *P* |
| **PSQI global score** |  |  |  |  | 0.88 | 0.46 |
| Baseline | 11.94(3.15) |  | 12.00(3.62) |  |  |  |
| Week 8 | 8.19(3.60) | 0.00 | 9.74(4.49) | 0.00 |  |  |
| Week 12 | 10.06(4.10) | 0.00 | 11.03(4.23) | 0.12 |  |  |
| Week 16 | 10.00(4.09) | 0.00 | 10.68(3.22) | 0.01 |  |  |
| Week 20 | 10.38(3.88) | 0.02 | 10.74(3.82) | 0.03 |  |  |
| **Duration of sleep** |  |  |  |  | 1.42 | 0.23 |
| Baseline | 1.97(0.82) |  | 2.03(0.91) |  |  |  |
| Week 8 | 1.31(0.86) | 0.00 | 1.61(1.02) | 0.045 |  |  |
| Week 12 | 1.81(0.90) | 0.42 | 1.87(0.96) | 0.34 |  |  |
| Week 16 | 1.72(0.89) | 0.13 | 1.71(0.97) | 0.06 |  |  |
| Week 20 | 1.91(1.03) | 0.75 | 1.65(0.95) | 0.03 |  |  |
| **Sleep** **disturbance** |  |  |  |  | 1.34 | 0.26 |
| Baseline | 1.09(0.30) |  | 1.00(0.00) |  |  |  |
| Week 8 | 1.00(0.25) | 0.18 | 1.03(0.18) | 0.33 |  |  |
| Week 12 | 1.00(0.00) | 0.08 | 1.00(0.00) | - |  |  |
| Week 16 | 1.06(0.25) | 0.66 | 1.03(0.18) | 0.33 |  |  |
| Week 20 | 1.00(0.00) | 0.08 | 1.00(0.00) | - |  |  |
| **Sleep** **latency** |  |  |  |  | 1.70 | 0.15 |
| Baseline | 2.12(1.10) |  | 1.74(1.37) |  |  |  |
| Week 8 | 1.41(1.16) | 0.01 | 1.74(1.32) | 1.00 |  |  |
| Week 12 | 1.94(1.27) | 0.44 | 1.97(1.22) | 0.21 |  |  |
| Week 16 | 2.12(1.07) | 1.00 | 2.06(1.18) | 0.11 |  |  |
| Week 20 | 2.09(1.06) | 0.88 | 2.10(1.14) | 0.09 |  |  |
| **Day dysfunctional due to sleepless** |  |  |  |  | 0.28 | 0.89 |
| Baseline | 1.97(0.86) |  | 1.81(0.87) |  |  |  |
| Week 8 | 1.25(0.92) | 0.00 | 1.23(0.99) | 0.01 |  |  |
| Week 12 | 1.44(1.08) | 0.02 | 1.32(1.11) | 0.04 |  |  |
| Week 16 | 1.47(1.16) | 0.051 | 1.16(0.97) | 0.00 |  |  |
| Week 20 | 1.44(1.05) | 0.02 | 1.23(1.02) | 0.02 |  |  |
| **Sleep** **efficiency** |  |  |  |  |  |  |
| Baseline | 1.87(0.94) |  | 2.10(1.01) |  | 0.82 | 0.99 |
| Week 8 | 1.44(0.91) | 0.046 | 1.61(1.20) | 0.02 |  |  |
| Week 12 | 1.81(0.97) | 0.73 | 1.90(1.01) | 0.23 |  |  |
| Week 16 | 1.59(1.04) | 0.14 | 1.71(0.94) | 0.06 |  |  |
| Week 20 | 1.84(1.05) | 0.88 | 1.97(1.11) | 0.50 |  |  |
| **Overall sleep** **quality** |  |  |  |  | 0.52 | 0.72 |
| Baseline | 1.88(0.79) |  | 1.84(0.69) |  |  |  |
| Week 8 | 1.34(0.60) | 0.00 | 1.29(0.59) | 0.00 |  |  |
| Week 12 | 1.50(0.62) | 0.02 | 1.65(0.71) | 0.08 |  |  |
| Week 16 | 1.53(0.72) | 0.054 | 1.61(0.67) | 0.09 |  |  |
| Week 20 | 1.66(0.75) | 0.20 | 1.58(0.62) | 0.04 |  |  |
| **Use of sleep medications** |  |  |  |  | 1.25 | 0.29 |
| Baseline | 1.03(1.33) |  | 1.48(1.44) |  |  |  |
| Week 8 | 0.44(1.01) | 0.01 | 1.23(1.41) | 0.16 |  |  |
| Week 12 | 0.56(1.13) | 0.01 | 1.32(1.45) | 0.36 |  |  |
| Week 16 | 0.50(1.11) | 0.01 | 1.39(1.48) | 0.52 |  |  |
| Week 20 | 0.41(1.01) | 0.00 | 1.23(1.48) | 0.06 |  |  |
